# Prevalence, pattern and determinants of disabilities in India: Insights from NFHS-5 (2019–21)

**DOI:** 10.3389/fpubh.2023.1036499

**Published:** 2023-02-27

**Authors:** Sweta Pattnaik, Jogesh Murmu, Ritik Agrawal, Tanveer Rehman, Srikanta Kanungo, Sanghamitra Pati

**Affiliations:** Department of Health Research, ICMR-Regional Medical Research Center, Bhubaneswar, Odisha, India

**Keywords:** disability, prevalence, NFHS-5, India, secondary data analysis

## Abstract

There is a need to provide an overview of the disability burden in India as there are limited studies. The present study aimed to estimate the prevalence and assess the pattern and determinants of disability in India. We analyzed National Family Health Survey-5 data using the “*svyset*” command in STATA software. We assessed the correlates by multivariable regression and reported an adjusted prevalence ratio (aPR) with a 95% confidence interval (CI). QGIS 3.2.1 software was used for spatial analysis of distributions of different disabilities. The mean (SD) age of 28,43,917 respondents was 30.82 (20.62) years, with 75.83% (*n* = 21,56,633) and 44.44% (*n* = 12,63,086) of them being from a rural area and were not educated, respectively. The overall prevalence of disability was 0.93% [(95% CI: 0.92–0.95), *n* = 26,435] and 5.11% of households have one or more people with disability (PwD). Locomotor disabilities accounted for 44.73% of all disabilities (*n* = 10,730), followed by mental disabilities (20.07%, *n* = 4,814). Age 75 years and above (vs. 0–14 years) [aPR: 26.35 (23.63–29.37)], male (vs. female) [aPR: 1.58 (1.52–1.64)], no education (vs. higher education) [aPR: 4.42 (4–4.87)], unmarried (vs. married) [aPR: 8.85 (8.27–9.47)], seeking care of non-governmental organization (NGO) (vs. other) [aPR: 1.34 (0.95–1.89)] were significant independent determinants. The highest overall prevalence of disability and locomotor was in Lakshadweep/UTs (1.68%) and Delhi (58.5%), respectively. Out of every hundred individuals in India, one has a disability, and five out of every hundred households have one or more people with a disability. More intervention strategies should be planned, considering factors like education, residence, health promotion and caste so that the services provided by the government can be available and accessible to everyone in need.

## Background

World Health Organization (WHO) defines disability as impairment, limitation, or restriction in activity caused mainly by health issues and environmental factors (1). Worldwide, about one billion (15%) individuals face some form of disability, and 20% have severe functional limitations (2). Since 80% of those with disabilities live in developing nations, it is essential to ensure their inclusion in all aspects of development (3).

Census 2011 and recently held 76^th^ round of the National Sample Survey (NSS) estimates the prevalence of disability was 2.2% in India (4, 5). Over 10 years, India's differently-abled population increased somewhat, growing from 21.9 to 26.8 million from 2001 to 2011, respectively (4). The prevalence of disabilities continues to rise gradually with age and is highest in individuals above 60 (6, 7). In India, by 2050, 323 million (19.1% of the total population) will be 60 years and above (8, 9). India will face significant structural and budgetary hurdles due to the increase in the aging population and people with disabilities.

Functional and physical disability is positively associated with co-existing chronic illnesses (7, 10). The interaction between chronic illness and physical disability has been explored (11). Non-communicable diseases (NCDs) like cardiovascular and musculoskeletal disorders account for 66.5% of disability-adjusted life years (DALYs) in low and middle-income countries (12, 13).

According to the 2019 Global Burden of Disease (GBD) report from 369 countries, road accidents accounted for nearly 5.1% DALYs among people aged 25 to 49. In contrast, ischemic heart disease and stroke were the leading causes of DALYs among people aged 50 to 75. Both contribute to shifting the burden due to “Years lived with disability” because of NCDs and injuries (14). India is undergoing an epidemiologic shift that increases the burden of NCDs (15). As of aging populations and changing lifestyles, NCDs are quickly expanding, the prevalence and likelihood of developing non-communicable diseases would increase exponentially, resulting in an increase in DALYs (16). Increased life expectancy is a result of demographic projection, which also causes a rise in chronic disease onset, that further adversely impacts people's overall health (17). This suggests that DALY is a comprehensive measurement that quantifies specific diseases and injuries in relation to NCD (18).

The “bio-psycho-social model” encompassing one's surroundings, personal attributes, quality of life, and self-sufficiency has shifted disability from a medical to a social model (19). International Classification of Functioning, Disability and Health (ICF) has classified disability into the hearing, visual, speech, mental and locomotor (1). The most common form in India is locomotor disability (20). Locomotor and hearing disabilities are significantly more common in Indian men than in Indian women (21).

A person with a disability (PwD) generally experiences adverse socioeconomic outcomes, poverty and physiologic stress, and inequity in access to essential resources such as education, health care facilities, employment, and social participation (22). Women with disability face challenges with access to reproductive and sexual health services and information (23). As India prepares for the next decennial census and in light of its pledge to the Committee on the Rights of Persons with Disability and Sustainable Development Goals (SDG), there is a need to provide an overview of the disability rates in India (24). Only a tiny portion of the PwD population in India receives government assistance (7). Moreover, there are limited studies that go into depth about the disability. For the first time, the fifth National Family Health Survey (NFHS) (2019–21) included five disability statuses to depict the burden of disability and its associated predictors. This study aimed to estimate the prevalence of disability in India, determine the associated factors and assess the pattern and geographical distribution using data from the most recent NFHS, 2019–2021.

## Methods

### Study setting

India is the world's second-most populous country (1.3 billion population), with 28 states and eight union territories (UTs). The Department of Empowerment of Persons with Disabilities (Divyangjan) was carved out of the Ministry of Social Justice and Empowerment in 2012 to ensure greater focus on policy matters and to address disability issues effectively. It acts as a nodal department for greater coordination among stakeholders, organizations, state governments and related central ministries. Consequently, the schemes have intended to increase accessibility through the supply of aids and assistive devices and educational and economic empowerment through skill development and financial assistance. India has eight national institutes and 20 composite regional centers, which provide services like early detection and intervention, counseling and medical rehabilitation to PwDs (25).

### Study design and study population

We conducted secondary data analysis on the NFHS-5 dataset. Initially, the proposal was submitted to Demographic Health Survey (DHS), after which authorization to use data was obtained. NFHS surveys capture data on the health and welfare of the Indian population through a nationally representative sample. We included all family members in the households surveyed. Transgender data were also provided, but we excluded them from the analysis due to their small population size (*n* = 180, 0.01%), which could lead to inconsistency in this study.

### Sample size and sampling technique

Villages and census enumeration blocks were chosen from districts in rural and urban areas, respectively, through a two-stage sampling procedure. Data collection was done using CAPI (Computer-assisted personal interview) from June 2019 to April 2021 with an inbuilt schedule and proper maintenance of confidentiality of respondents' answers. NFHS-5 methodology, including selecting households and data collection procedures, has been meticulously described and published elsewhere (26). The questionnaire was administered to the head of the family, and a total of 28,43,917 participants of all age groups were included in our study.

### Data variables and data sources

The independent variables for assessing the prevalence of disability were sociodemographic and health-seeking behaviors characteristics. Some of the covariates are age (categorized into 0–14, 15–29, 30–44, 45–59, 60–74, and 75 years and above); marital status classified as “married” (those who are currently married), “formerly/ever married” (previously ever married including divorced, widowed, not living together, separated), and “unmarried (never married)”; education according to completed years of schooling (“no education”- those who had no formal schooling, “up to primary”- < 5 years of education, “up to secondary”- 5–9 years, “higher” > 10 years); Below Poverty Level (BPL) card holder; health-seeking behavior (public, private, non-governmental organization (NGO)/trust hospitals/clinics, and others-which included those who sought treatment from pharmacy outlets, home treatment, and treatment from any other source). In NFHS-5, disability was considered present if the participant responded “yes” to the question: “If any household member, including you, have any disability?” Out of those identified as “disability present,” it was further classified into sub-categories “Hearing,” “Speech,” “Visual,” “Mental,” “Locomotor.”

### Operational definitions

We have given the operational definitions of various types of disability as per the information provided in the NFHS-5 report in Supplementary File 1.

### Statistical analysis

STATA 16 (Stata Corp, College Station, Texas, USA) was used for statistical analysis. Before analyzing, all flagged, missing, and no information cases were removed while recording variables. The NFHS sampling weights were used to justify the differential probabilities of participant selection and ensure the validity of our study findings. The “*svyset*” command was used to declare the dataset as survey type and to estimate the population's weighted proportion. The burden of disability and its predictors were estimated using the weighted prevalence and reported with a 95% confidence interval (CI). Univariate log-binomial regression was done for all the independent variables with the outcome and reported an unadjusted prevalence ratio (PR) with 95% CI. Other form of disabilities was not specified under the heading “Others” in the categories of disabilities because different types of disabilities were not clearly mentioned in the NFHS-5 dataset. Therefore, they were excluded from the table of types of disabilities, giving a total number of persons with disability (*n* = 2,447).

Consequently, multivariable regression was done after checking for collinearity among the variables using the variance inflation factor and reported adjusted PR with 95% CI. Variables with *p* < 0.05 were considered significant. To determine the regional differences in disabilities, we have assessed the overall prevalence of disabilities; along with it, we have shown the nationwide prevalence of the three most prevalent disabilities as per the current study, i.e., locomotor, mental, followed by speech. We have used QGIS 3.2.1 software (Available from: http://qgis.osgeo.org) (27). To make it nationally representative, we have used weighted data for our analysis.

### Ethical consideration

There is no risk to participants because the current study is based on secondary, anonymized data obtained from DHS. Informed consent for all the respondents was obtained during the survey. The dataset used is duly acknowledged and cited wherever needed. This study has been scrutinized and declared for exemption for review by IEC as there is less than minimal risk and no linked identifiers bearing Ref: …ICMR-RMRC/IHEC-2022/150.

## Results

The analysis includes a total of 2,843,917 respondents of all age groups. The respondents' mean (SD) age was 30.82 ± 20.62 years. Of the total, 26.92% were between the ages of 0 and 14 years (*n* = 765,602), 50.41% were females (*n* = 1,433,580), 75.83% belonged to rural residents (*n* = 2,156,633), and 49.99% were married (*n* = 1,421,809) (Table 1).

**Table 1:** Sociodemographic and health-seeking behavioral characteristics of the study population covered in NFHS-5 (*N* = 28,43,917).

**Characteristics**	**Categories**	Frequency (*n*, %^*^)	Weighted frequency (*n*, %^*^)
Age	0–14 years	7,65,602 (26.92)	7,53,584 (26.50)
	15–29 years	7,39,990 (26.02)	7,42,061(26.09)
	30–44 years	5,73,971 (20.18)	5,73,200 (20.16)
	45–59 years	4,40, 751 (15.50)	4,41,851 (15.54)
	60–74 years	2,61,321 (9.19)	2,69,714 (9.48)
	75 years and above	62,843 (2.19)	63,506 (2.23)
Gender (*N* = 28,43,734)^†^	Male	14,10,154 (49.59)	14,07,502 (49.49)
	Female	14,33,580 (50.41)	14,36,232 (50.51)
Residence	Urban	6,87,284 (24.17)	9,00,407 (31.66)
	Rural	21,56,633 (75.83)	19,43,510 (68.34)
Educational status (*N* = 28,42,431)^†^	No education	12,63,086 (44.44)	12,36,658 (43.51)
	Primary	11,08,398 (38.99)	11,01,206 (38.74)
	Secondary	1,96,536 (6.91)	1,94,948 (6.86)
	Higher	2,74,411 (9.65)	309,619 (10.89)
Marital status	Unmarried	12,50,853 (43.98)	12,28,826 (43.21)
	Married	14,21,809 (49.99)	14,39,883 (50.63)
	Formerly/ever married	1,71,255 (6.02)	1,75,208 (6.16)
Region	North	5,83,110 (20.50)	3,99,373 (14.04)
	Central	6,86,111 (24.13)	7,21,765 (25.38)
	East	4,66,522 (16.40)	6,40,383 (22.52)
	North-east	3,91,078 (13.75)	1,01,557 (3.57)
	West	2,89,723 (10.19)	4,13,100 (14.53)
	South	4,27,373 (15.03)	5,67,738 (19.96)
Religion	Hinduism	21,38,965 (75.21)	23,04,244 (81.02)
	Islam	3,62,313 (12.74)	3,88,621 (13.66)
	Christianity	2,02,918 (7.14)	68,564 (2.41)
	Others	1,39,721 (4.91)	82,489 (2.90)
Caste	Scheduled caste	5,59,048 (19.66)	6,23,405 (21.92)
	Scheduled tribe	5,31,496 (18.69)	2,69,776 (9.49)
	Other backward class	10,60,884 (37.30)	11,91,536 (42.04)
	Other	6,92,489 (24.35)	7,55,200 (26.55)
Wealth index	Poorest	6,36,437 (22.38)	5,69,605 (20.03)
	Poorer	6,28,147 (22.09)	5,69,983 (20.04)
	Middle	5,75,696 (20.24)	5,69,127 (20.01)
	Richer	5,24,896 (18.46)	5,68,180 (19.98)
	Richest	4,78,741 (16.83)	5,67,022 (19.94)
Health insurance scheme (*N* = 28,29,625)^†^	Absent	16,22,398 (57.34)	16,82,783 (59.47)
	Present	12,07,227 (42.66)	11,46,842 (40.53)
BPL holder (*N* = 28,39,275)^†^	No	14,49,238 (51.04)	15,47,900 (54.52)
	Yes	13,90,037 (48.96)	12,91,375 (45.48)
Seek healthcare preferably at which treatment facility	Public facility	16,13,875 (56.75)	13,83,735 (48.66)
	Private facility	11,86,216 (41.71)	14,11,897 (49.65)
	NGO/Trust	10,502 (0.37)	13,288 (0.47)
	Other	33,324 (1.17)	34,997 (1.23)

BPL, Below Poverty Level; NGO, Non-government organization.

^*^Column percentage,

^†^missing and no information participants were removed.

The overall prevalence of disability was 0.93% [(95% CI: 0.92–0.95), *n* = 26,435] and 5.11% of households have one or more people with disability (PwD) across all age groups in India. The prevalence was highest in the age group of 75 years and above at 1.96% (Table 2).

**Table 2:** Determinants of disability in the study population covered in NFHS-5 (*N* = 2,843,917).

**Characteristics**	**Disability**		**Univariable regression**	**Multivariable regression**	
	***n***	**%** ^*^ **, 95% CI**	**PR, 95% CI**	**aPR, 95% CI**	***p*** **-value**
**Age of participant** ^†^					
0–14 years	4,043	0.53 (0.52–0.55)	Reference	Reference	
15–29 years	6,400	0.86 (0.84–0.88)	1.61 (1.53–1.70)	5.23 (4.87–5.63)	< 0.001
30–44 years	6,440	1.12 (1.09–1.15)	2.11 (1.99–2.23)	21.47 (19.75–23.33)	< 0.001
45–59 years	4,780	1.08 (1.05–1.11)	2.03 (1.91–2.82)	19.89 (18.21–21.71)	< 0.001
60–74 years	3,803	1.41 (1.36–1.45)	2.65 (2.49–2.82)	22.22 (20.33–24.28)	< 0.001
75 and above	1,243	1.96 (1.85–2.07)	3.70 (3.40–4.03)	26.35 (23.63–29.37)	< 0.001
**Gender (*****N*** = **2,843,734)**^†^					
Male	16,054	1.14 (1.12–1.16)	1.54 (1.49–1.60)	1.58 (1.52–1.64)	< 0.001
Female	10,655	0.74 (0.73–0.76)	Reference	Reference	
**Residence**					
Urban	7,623	0.85 (0.82–0.87)	Reference	Reference	
Rural	19,087	0.98 (0.96–0.99)	1.61 (1.11–1.21)	0.98 (0.9–1.02)	0.369
**Education (*****N*** = **2,842,431)**^†^					
No education	14,761	1.19 (1.17–1.21)	2.37 (2.20–2.55)	4.42 (4–4.87)	< 0.001
Primary	9,225	0.84 (0.82–0.85)	1.65 (1.53–1.79)	2.06 (1.90–2.25)	< 0.001
Secondary	1,134	0.58 (0.55–0.61)	1.14 (1.03–1.27)	1.21 (1.09–1.36)	< 0.001
Higher	1,569	0.51 (0.48–0.53)	Reference	Reference	
**Marital status** ^†^					
Unmarried	12,771	1.04 (1.02–1.06)	1.33 (1.28–1.38)	8.85 (8.27–9.47)	< 0.001
Married	11,255	0.78 (0.76–0.79)	Reference	Reference	
Formerly/ever married	2,684	1.53 (1.47–1.59)	1.97 (1.86–2.09)	1.37 (1.28–1.46)	< 0.001
**Region** ^†^					
North	1,988	0.87 (0.83–0.90)	1.08 (1.01–1.15)	1.38 (1.28–1.48)	< 0.001
Central	7,265	0.81 (0.79–0.83)	1.01 (0.95–1.07)	1.20 (1.12–1.28)	< 0.001
East	5,829	0.91 (0.88–0.93)	1.13 (1.06–1.21)	1.22 (1.13–1.31)	< 0.001
North-east	815	0.80 (0.74–0.85)	Reference	Reference	
West	4,407	1.07 (1.03–1.09)	1.33 (1.23–1.43)	1.67 (1.55–1.81)	< 0.001
South	6,405	1.13 (1.10–1.15)	1.41 (1.32–1.50)	1.66 (1.55–1.78)	< 0.001
**Religion** ^†^					
Hinduism	21,615	0.94 (.092–0.95)	0.92 (0.83–1.02)	0.93 (0.83–1.03)	0.175
Islam	3,462	0.89 (0.86–0.92)	0.87 (0.78–0.98)	0.84 (0.75–0.95)	0.006
Christianity	698	1.02 (0.94–1.09)	Reference	Reference	
Others	934	1.13 (1.06–1.20)	1.11 (0.98–1.27)	1.20 (1.32–1.51)	0.009
**Caste** ^†^					
Scheduled caste	6,164	0.99 (0.96–1.01)	1.13 (1.07–1.20)	1.27 (1.19–1.35)	< 0.001
Scheduled tribe	2,354	0.87 (0.83–0.90)	Reference	Reference	
Other backward class	11,361	0.95 (0.93–0.97)	1.08 (1.03–1.15)	1.35 (1.28–1.43)	< 0.001
Other	6,831	0.90 (0.88–0.92)	1.03 (0.97–1.10)	1.41 (1.32–1.19)	< 0.001
**Wealth index** ^†^					
Poorest	6,574	1.15 (1.12–1.18)	1.77 (1.67–1.88)	1.55 (1.43–1.68)	< 0.001
Poorer	6,044	1.06 (1.03–1.08)	1.62 (1.53–1.73)	1.38 (1.28–1.48)	< 0.001
Middle	5,595	0.98 (0.95–1.00)	1.51 (1.41–1.61)	1.24 (1.16–1.33)	< 0.001
Richer	4,789	0.84 (0.82–0.07)	1.29 (1.21–1.38)	1.11 (1.04–1.19)	0.002
Richest	3,707	0.65 (0.63–0.67)	Reference	Reference	
**Health insurance scheme (*****N*** = **2,829,625)**					
Absent	15,386	0.91 (0.89–0.93)	Reference	Reference	
Present	11,203	0.98 (0.95–1.00)	1.07 (1.03–1.10)	1.02 (0.98–1.06)	0.188
**BPL holder (*****N*** = **2,839,275)**^†^					
No	12,784	0.83 (0.81–0.84)	Reference	Reference	
Yes	13,877	1.07 (1.05–1.09)	1.30 (1.26–1.35)	1.08 (1.04–1.12)	< 0.001
**Treatment facility** ^†^					
Public facility	65,717	4.75 (4.71–4.78)	1.21 (1.05–1.40)	1.22 (1.05–1.42)	0.007
Private facility	60,767	4.30 (4.27–4.33)	1.00 (0.86–1.16)	1.08 (0.93–1.26)	0.281
NGO/Trust	662	4.98 (4.62–5.36)	1.30 (0.93–1.83)	1.34 (0.95–1.89)	0.089
Other	1,380	3.94 (3.74–4.15)	Reference	Reference	

PR, prevalence ratio; aPR, adjusted prevalence ratio; CI, confidence interval; BPL, below poverty level; NGO, non-government organization.

^*^Row percentage.

†p-value < 0.05.

Respondents aged 75 years and above had twenty-six [aPR: 26.35 (23.63–29.37)] the prevalence of disability compared with 0–14 years (Table 2). Disability was 58% more among males [aPR: 1.58 (1.52–1.64)] than females. Regarding education, disability was four times more common among those who didn't have any form of schooling [aPR: 4.42 (4–4.87)] in contrast to those who have completed higher education. Unmarried people had eight times more disability [aPR: 8.85 (8.27–9.47)] than married people. Respondents belonging to the west region [aPR: 1.67 (1.55–1.81)] have 67% more prevalence of disability compared with the north-east region. People from other backward castes had a 35% more burden of disability compared to people from scheduled tribe [aPR: 1.35 (1.28–1.43)]. Disability was 55% higher in the poorest wealth quintile [aPR: 1.55 (1.43–1.68)] than in most affluent. Individuals with disabilities favored NGOs or Trust hospitals/clinics for medical care [aPR: 1.34 (0.95–1.89)] over visiting pharmacies or taking home treatment.

**Figure 1:**
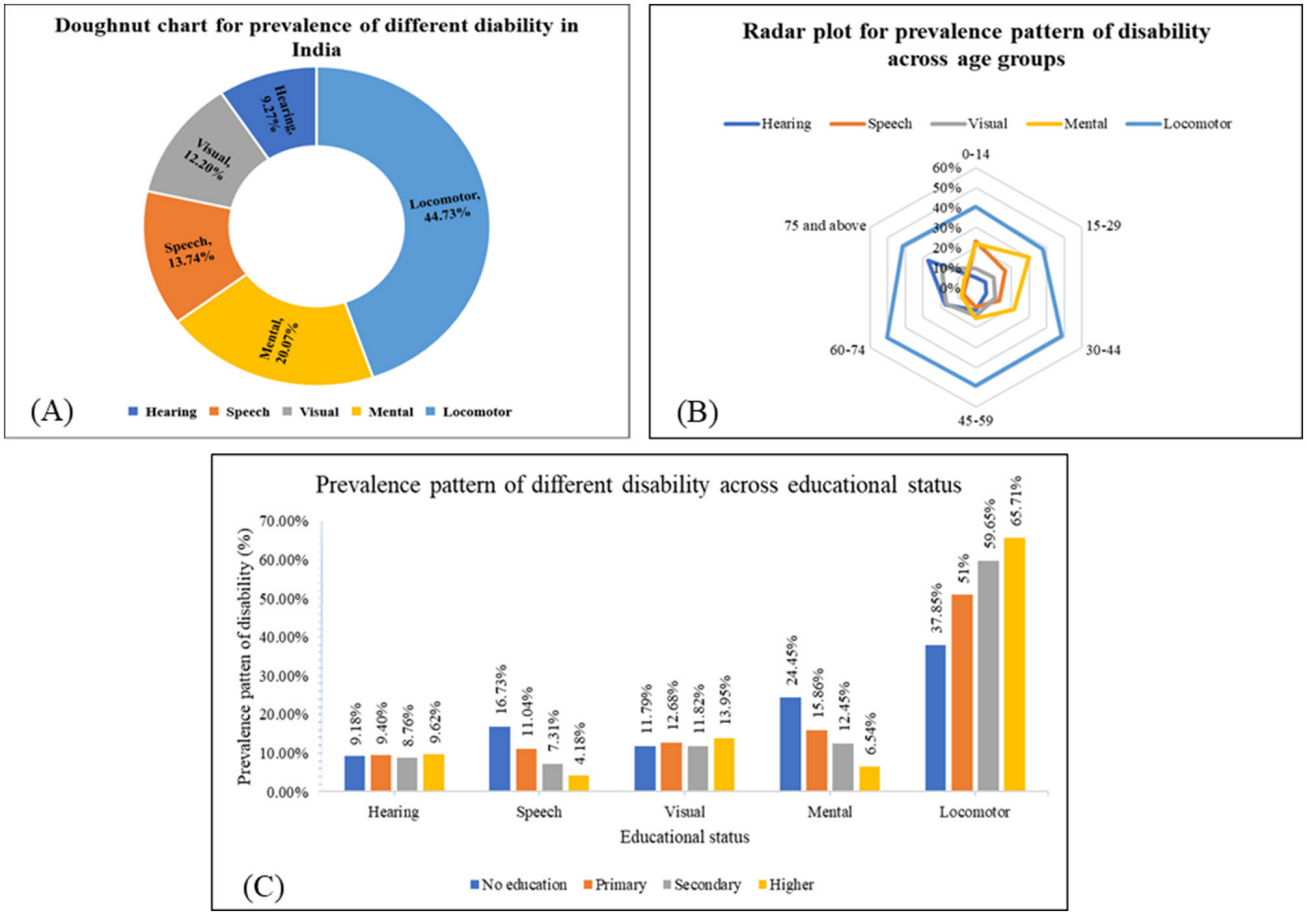
Prevalence of different disabilities across sociodemographic characteristics. **(A)** Doughnut chart for the prevalence of different disabilities across the population in India based on NFHS-5 (*N* = 23,988). **(B)** Radar plot showing the prevalence pattern of different disabilities across age groups in India based on NFHS-5 (*N* = 23,988). **(C)** The prevalence pattern of different disabilities across educational statuses in India based on NFHS-5 (*N* = 23,988).

Of the total, locomotor disabilities accounted for 44.73% [(95% CI: 43.87–45.59), *n* = 10,730] followed by mental [20.07% (95% CI: 19.38–20.77), *n* = 4,814] and speech disabilities [13.74% (95% CI: 13.14–14.35, *n* = 3,295; Figure 1A). The detailed prevalence of individual disabilities is given in Supplementary File 2. The ages of 60–74, 15–29, and 0–14 years had the highest burden of locomotor disability (50.47%), mental disability (29.98%), and speech disability (23.06%) respectively.

The preponderance of locomotor disability is highest among the 60–74 years age group. The prevalence pattern of various disabilities across the age groups is shown in Figure 1B.

The detailed prevalence pattern of various disabilities across educational status is shown in Figure 1C. Higher educational attainment is associated with a higher prevalence of locomotor and visual disabilities, as well as a lower prevalence of mental and speech disabilities.

**Figure 2:**
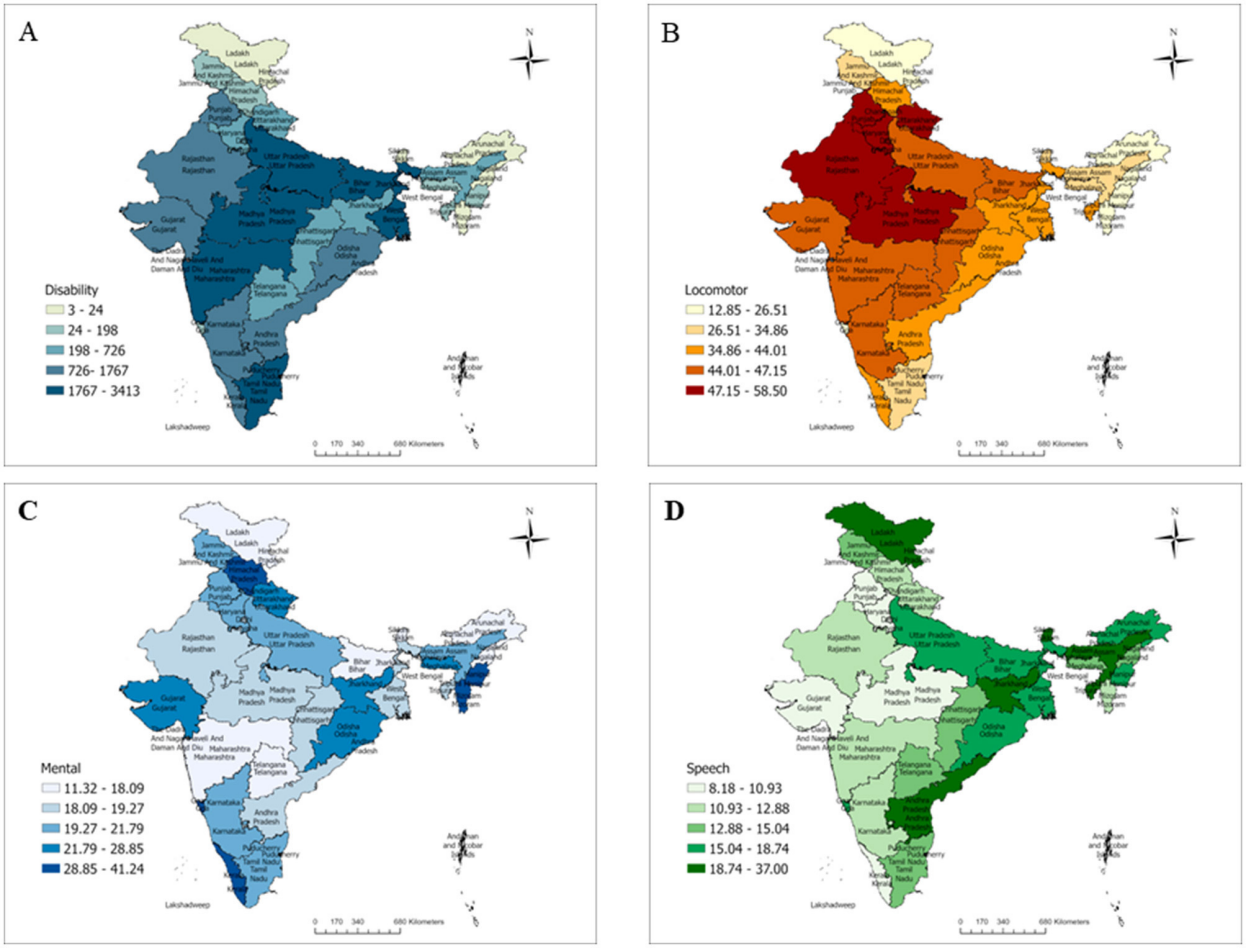
Prevalence patterns of disability in India based on NFHS-5. **(A)** Overall prevalence pattern of disability in India, NFHS 5. **(B)** Distribution of locomotor disability in India, NFHS 5. **(C)** Distribution of mental disability in India, NFHS 5. **(D)** Distribution of speech disability in India, NFHS 5.

Figures 2A–D shows the burden of disability and its pattern across the states and UTs of India. The overall disability distribution given in Figure 2A indicates that it is more prevalent in Lakshadweep, UT (1.68%), followed by Tamil Nadu (1.26%) and Karnataka (1.22%). In the present study, the regional disparities could be because of the fact that composition of the population and the individuals with disability varies in different states. So, the prevalence of disability varies in different states and found to be higher in Lakshadweep where the total population is less as compared with other states and UTs. For national representativeness, we have used the weighted values for data. Similarly, the prevalence of locomotor disability (Figure 2B) was highest in Delhi (58.5%), followed by Punjab (55.51%) and Madhya Pradesh (53.47%). Figure 2C shows the prevalence of mental disabilities, with the highest in Lakshadweep (41.24%), followed by Mizoram (38.12%) and Goa (37.1%). Figure 2D shows the highest prevalence of speech disability in Sikkim (37%), followed by Tripura (22.66%) and Jharkhand (22.12%).

## Discussion

The overall prevalence of disability in India based on secondary data analysis of the NFHS-5 survey (2019–2021) was 0.93% and 5.11% of households have one or more PwDs. Locomotor disabilities accounted for 44.73% of all disabilities, followed by mental and speech disabilities. The highest prevalence of locomotor, mental, and speech disability was in Delhi, Lakshadweep, and Sikkim, respectively, whereas the overall prevalence was highest in Lakshadweep/UTs.

In the present study, the prevalence of disability was found to be 0.93%, with 5.11% of households including one or more PwDs. While our study shows a notably lower overall prevalence of disability compared to countries like Myanmar (4.6%) and South Africa (4.9%) (28, 29), the household prevalence of PwDs is comparable or even higher. For instance, despite lower overall prevalence rates, the household prevalence in our study exceeds that reported in countries such as Zimbabwe (2.9%) and Cambodia (4%), and is similar to, or even higher than, the rates observed in countries like Jordan (13%) and Zimbabwe (7%) at the household level (30, 31). The burden of disability varies country-wise. Most surveys conducted in developed countries concentrated on wider spheres of participation and the need for services. However, most surveys done in LMICs typically emphasize impairment questions. The dynamic interaction between health, environmental, and personal contexts that vary among regions contributes to the occurrence of disabilities (32). Also, sampling technique, type of population involved, sociodemographic characteristics, and population composition varies.

Our finding suggests a higher prevalence of locomotor disability, which is higher than the study conducted in Mumbai (5.57%) (33). The present study highlights that locomotor disability was 286 highest among those aged 60–74 years (34). Although there is limited evidence supporting this, the most likely cause could be the rising prevalence of both acquired and congenital locomotor disability, including rickets, tuberculosis spine, and clubfoot (congenital talipes equinovarus or CTEV) (35).

Age is a significant predictor of disability and is positively associated with disability. The study by Gupta et al. (36) revealed that the prevalence of disability increases with age, with the highest in the age group of 75 years and above (63.8%), which is in harmony with the results of the current study (36). Degenerative health conditions (such as arthritis and spondylosis), chronic health conditions, falls, and injuries are some factors that increase the susceptibility to disability among older age groups (37). Another study found that in the 60–64 age group, only 36% have some disability, and 61% are 75 years and above (36). The difference in this result from the present study could be because of the difference in scales used in disability. And older adults are at high risk of developing intellectual and neurological disorders or substance use problems and are vulnerable to other health-related conditions such as hearing loss and osteoarthritis (38). In older adults, aging causes a variety of psychological issues, which includes: (1) reduced proprioception, (2) diminished ability to adapt to environmental changes, social roles and status, (3) elevated risk of exposure to adverse life effects such as retirement from a job, financial management and death of relative (39).

Our findings suggest that men are more prone to have any disability as compared to women. However, women aged 65–79 years are 3.3% more likely to have functional limitations than men, and with an increase in age of 80 years or older the likelihood of disability increases to 15.5% (40). Despite an increase in the prevalence of disability with age progression, female dominance is seen, which contrasts with our study findings. As a result, it shows an apparent gender disparity in disability prevalence estimates rates. A community-based study in rural Haryana shows that functional disability was lower in men (35.9%) as compared with women (38.8%), which is also a contrast to our findings (36). This disparity may be because males are more likely to encounter accidents and injuries and risk developing NCDs (41). Males' participation in risky activities and physically demanding occupations (mine, electrical and telecommunications, climbing and working, commercial driving, and so on) could also be a possible explanation.

The study by Yadav and Singh (42) suggested that adults between the ages of 20 and 25 had a higher prevalence of disability than children between the ages of 10 and 14. Adults may have a higher prevalence of NCDs due to increased risk of road traffic accidents (RTAs), self-harm, and behavioral changes like alcoholism, tobacco use, and drug abuse (42).

Our study observed that the disability was higher among those with lesser years of schooling. Most disabled people were undereducated, as shown by the study from southwest Turkey and in China among community dwellers, including older individuals, in harmony with our study findings (43, 44). Even though the government mandates a 5% reservation in government-aided institutions and a 4% reserve in government jobs, the prevalence of disability is higher among those with lower levels of education (45). These could result from difficulties related to attitude, a lack of inclusivity, transportation issues, and parents' and caregivers' lack of understanding of the importance of obtaining education for their kids (46). Additionally, prior research has shown that the lowest wealth quintile had a much higher risk of death and disability than their highest wealth counterparts at all ages, which is similar to our current study's finding. Wealth may be a better predictor of scarce financial resources, exacerbated by a loss of employment, retirement, or advancing age (47). People living in poverty may work under hazardous conditions associated with adverse health outcomes, including disability. They may also have limited access to healthcare and education, which puts them at a greater risk of developing disabilities (10, 48).

A study shows that formerly/ever-married and unmarried people tend to suffer more from functional limitations, which is in line with our findings. This observation is also validated by a more comprehensive survey of 57 countries worldwide (49, 50). PwD (cognitive impairment or mobility difficulties) may appeal less to potential partners due to partner selection and independent choices. According to one study, men refuse to marry disabled women despite their awareness of their stigma and discrimination. They desire spouses who can give the physical support they require while overcoming considerable obstacles to fulfilling their roles as a husband, father, and provider (51). This also can result from the spouse abandoning their disabled partner, who can no longer support them as a couple (52).

The prevalence of disability also varies according to region. Topographically the southern part was found to be a potential domain for disability in our study. A regional assessment of disability in India revealed that the country's central zone has the highest percentage of total disability, which contrasts with our findings (53). Despite notable advancement in the health index score in a report by NITI Aayog (National Institution for Transforming India) in western states like Maharashtra and Gujarat, the rates of disability were higher in this study (54). However, the level of healthcare infrastructure is not improving in states such as Rajasthan, which has a low health index score. According to a study, most disabled people in Rajasthan and Gujarat receive treatment after the onset of their disability (55). It could be due to the level of services and facilities the state provides, with uncrowded hospitals providing better medical facilities and treatments.

The study conducted in Chennai among minorities suggested that rates of disability were higher among those belonging to Scheduled Tribes and Scheduled Castes (STs and SCs). In contrast, our study found that disability was more prevalent among individuals belonging to Scheduled Castes (SCs) and Other Backward Classes (OBCs) (56). These communities continue to face economic discrimination and societal violence in many parts of the country, which frequently leads to violence resulting in the death or injury of victims suggestive of the occurrence of any disability (57).

Our analysis reveals that most people with disabilities have health insurance (58). The Indian government has made provisions for various health insurance schemes for people with disabilities. Two are the Niramaya Health Insurance Scheme and the Swavlamban Health Insurance Scheme. Whilst the latter was discontinued for unspecified reasons, the former provides beneficiaries affordable health insurance (around INR 1 lakh plus additional services) (58, 59). Other initiatives that work for the betterment of PWDs include the Deendayal Disabled Rehabilitation Scheme (DDRS), Sugamya Bharat Abhiyan, Assistance to Disabled Persons for Purchase / Fitting of Aids / Appliances (ADIP), and the Unique Disability ID Project (UDID). Volunteers' proactive participation, extensive collaboration with NGOs, and comprehensive publicity will draw more public attention to these schemes (60). In Bangladesh, a cross-sectional study reported that most participants visited private clinics or hospitals; however, in our research, we discovered that most people with disabilities chose to obtain medical care from NGOs or Trusts, possibly because it was less expensive than going to the private hospital, good quality of care, less waiting time, limited government facility nearby and provision of ancillary services like assistive device (24, 61).

We have also estimated the prevalence and patterns of various types of disabilities (Hearing, Speech, Visual, Mental, and Locomotor) across different sociodemographic statuses, access, and quality of health services that influence the health and wellbeing of the population (62). Consistent with previous research, the current study shows disparities in the prevalence of disability types by age, gender, educational status, region, wealth index, caste, and treatment facility (63).

### Policy implications

When we look into the interrelationship between disability and covariates, we find that education is strongly linked with disability. There is a need to shift the emphasis toward health education through Information, Education and Communication (IEC) and Behavior Change Communication (BCC) strategies. The RPWD Act 2016 is a fully-fledged initiative by the Indian government to guarantee equitable services. Community-based rehabilitation (CBR) is an essential component of this strategy. However, a low CBR to PWDs ratio, limited resources, and cultural preferences impede programmes' efficiency. Understanding the burden of disability will be made easier with the help of the recruitment of professionally trained personnel, resource allocation, logistical management, and a CBR database. Despite the government's ongoing efforts, a gap still needs to be bridged. Discrimination, inequality, and social difficulties are still persistent problems. The existing gap can be filled through active education and distribution of disability, how it can be managed, and how it does not make a difference in a society. Dissemination of disability-related initiatives, encouragement of the value of education among those with congenital disabilities, and methods of vocational employment at the grassroots level would be beneficial. Accredited Social Health Activist training and sensitization on raising awareness about disability and discussing it with families will significantly impact it.

### Strength and limitations

To the best of our knowledge, this is the first study to estimate the prevalence and determinants of disability across households in India. Because the study is based on nationally representative data from a household survey, it ensures generalizability regarding the prevalence of various disabilities. However, the cross-sectional nature of this study allows it to consider the self-reported incidents as described by the respondents. It can be challenging to assess whether a person has an impairment since it is sometimes in the latent phase, making it difficult to diagnose and hence vulnerable to bias. This study has a few more limitations since the certificate of the disabled respondents has not been checked, and more than one disability is not given separately in the dataset. Furthermore, we have not considered NCD as data were unavailable.

## Conclusion

The overall prevalence of disability in India is 0.93% and 5.11% of households have one or more people with disability (PwD). Locomotor disability is the most common type of disability among the population. More intervention strategies should be planned, considering factors like education, health promotion and caste so that the services provided by the government can be available and accessible to everyone in need.

## Data availability statement

Publicly available datasets were analyzed in this study. This data can be found at: https://www.dhsprogram.com/methodology/survey/survey-display-541.cfm.

## Author contributions

SPati, SK, and TR: concept and design. SPati, SK, and TR: monitored analysis and critical revision of the manuscript for important intellectual content. SPati, SK, and TR: administrative and technical support. JM, SPatt, and RA: acquisition, statistical analysis or interpretation of data, and drafting of the manuscript. SPati: supervision. All authors reviewed the manuscript.
