# Corrigendum: Prevalence, pattern, and determinants of disabilities in India: Insights from NFHS-5 (2019–21)

**DOI:** 10.3389/fpubh.2024.1487631

**Published:** 2024-11-01

**Authors:** Sweta Pattnaik, Jogesh Murmu, Ritik Agrawal, Tanveer Rehman, Srikanta Kanungo, Sanghamitra Pati

**Affiliations:** Department of Health Research, ICMR-Regional Medical Research Center, Bhubaneswar, Odisha, India

**Keywords:** disability, prevalence, NFHS-5, India, secondary data analysis

In the published article, there was an error in Figures 1A–C, and 2A–D, and Table 2 as published.

Due to a change in the data, the percentage, prevalence, confidence intervals (CI), adjusted prevalence ratios (aPR), and percentages have been revised in the tables and figures.

The corrected Figures 1, 2, and Table 2 and their captions appear below.

**Figure 1:**
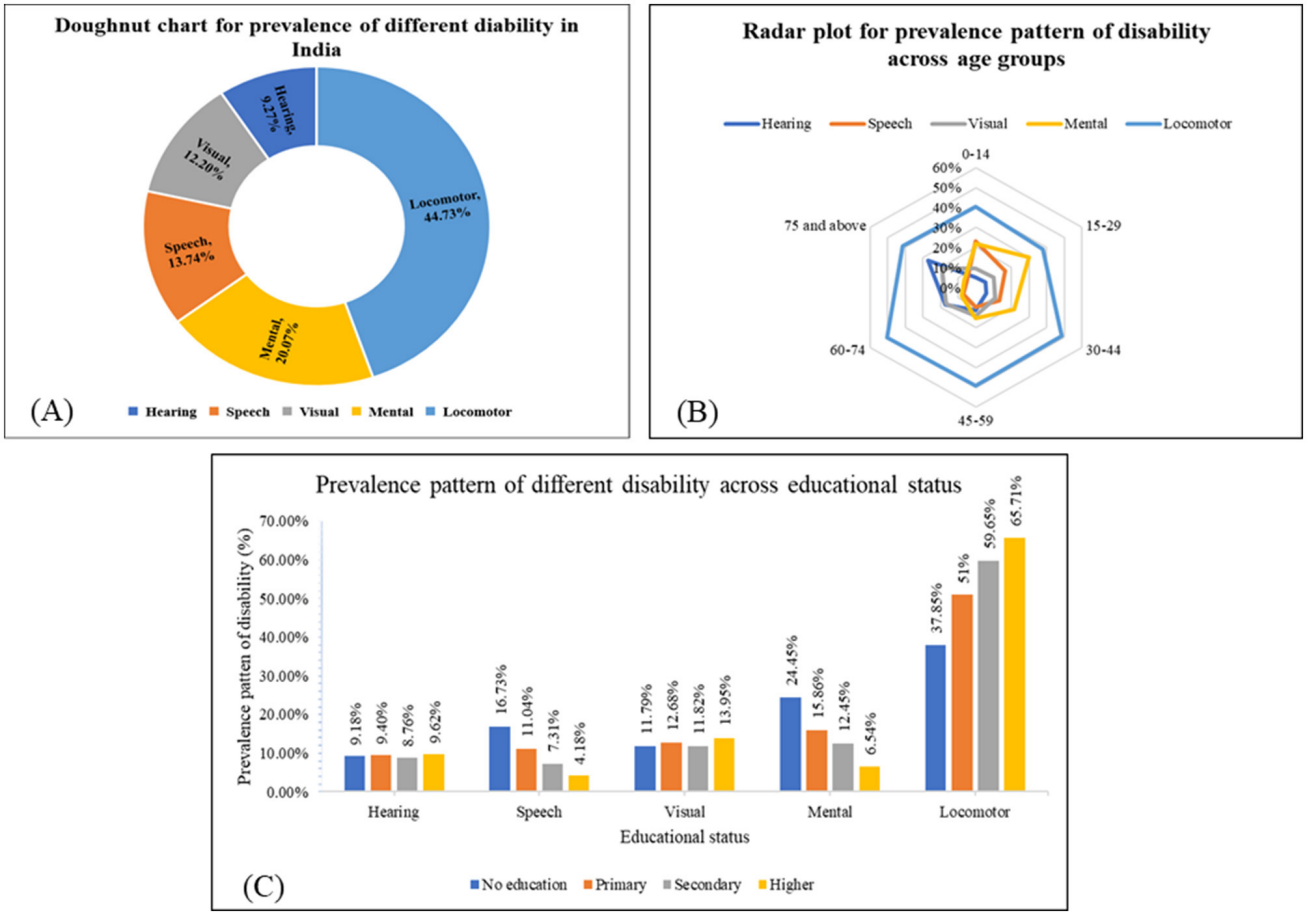
Prevalence of different disabilities across sociodemographic characteristics. **(A)** Doughnut chart for the prevalence of different disabilities across the population in India based on NFHS-5 (*N* = 23,988). **(B)** Radar plot showing the prevalence pattern of different disabilities across age groups in India based on NFHS-5 (*N* = 23,988). **(C)** The prevalence pattern of different disabilities across educational statuses in India based on NFHS-5 (*N* = 23,988).

**Figure 2:**
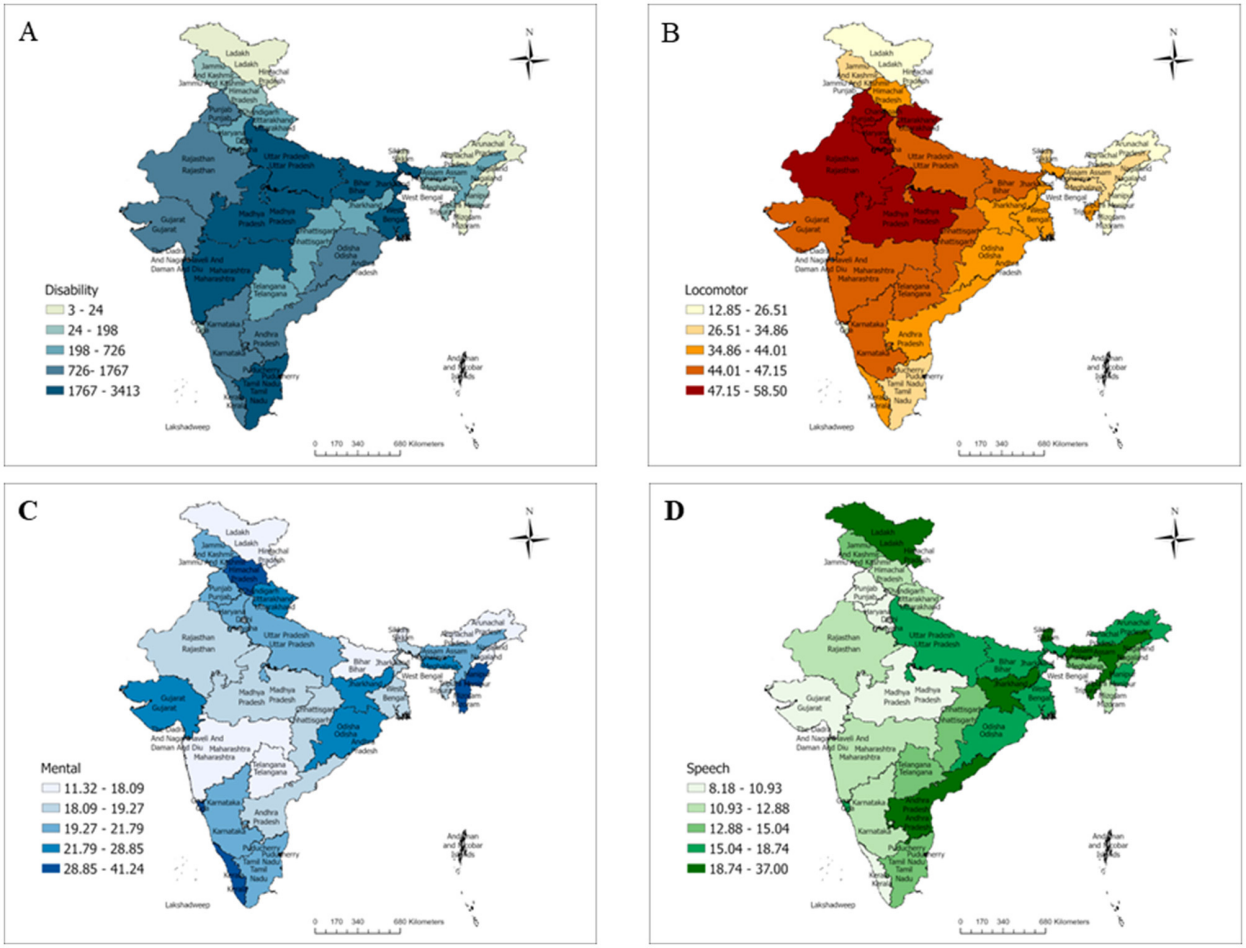
Prevalence patterns of disability in India based on NFHS-5. **(A)** Overall prevalence pattern of disability in India, NFHS 5. **(B)** Distribution of locomotor disability in India, NFHS 5. **(C)** Distribution of mental disability in India, NFHS 5. **(D)** Distribution of speech disability in India, NFHS 5.

**Table 2:** Determinants of disability in the study population covered in NFHS-5 (*N* = 2,843,917).

**Characteristics**	**Disability**		**Univariable regression**	**Multivariable regression**	
	***n***	**%** ^*^ **, 95% CI**	**PR, 95% CI**	**aPR, 95% CI**	***p*** **-value**
**Age of participant** ^†^					
0–14 years	4,043	0.53 (0.52–0.55)	Reference	Reference	
15–29 years	6,400	0.86 (0.84–0.88)	1.61 (1.53–1.70)	5.23 (4.87–5.63)	< 0.001
30–44 years	6,440	1.12 (1.09–1.15)	2.11 (1.99–2.23)	21.47 (19.75–23.33)	< 0.001
45–59 years	4,780	1.08 (1.05–1.11)	2.03 (1.91–2.82)	19.89 (18.21–21.71)	< 0.001
60–74 years	3,803	1.41 (1.36–1.45)	2.65 (2.49–2.82)	22.22 (20.33–24.28)	< 0.001
75 and above	1,243	1.96 (1.85–2.07)	3.70 (3.40–4.03)	26.35 (23.63–29.37)	< 0.001
**Gender (*****N*** = **2,843,734)**^†^					
Male	16,054	1.14 (1.12–1.16)	1.54 (1.49–1.60)	1.58 (1.52–1.64)	< 0.001
Female	10,655	0.74 (0.73–0.76)	Reference	Reference	
**Residence**					
Urban	7,623	0.85 (0.82–0.87)	Reference	Reference	
Rural	19,087	0.98 (0.96–0.99)	1.61 (1.11–1.21)	0.98 (0.9–1.02)	0.369
**Education (*****N*** = **2,842,431)**^†^					
No education	14,761	1.19 (1.17–1.21)	2.37 (2.20–2.55)	4.42 (4–4.87)	< 0.001
Primary	9,225	0.84 (0.82–0.85)	1.65 (1.53–1.79)	2.06 (1.90–2.25)	< 0.001
Secondary	1,134	0.58 (0.55–0.61)	1.14 (1.03–1.27)	1.21 (1.09–1.36)	< 0.001
Higher	1,569	0.51 (0.48–0.53)	Reference	Reference	
**Marital status** ^†^					
Unmarried	12,771	1.04 (1.02–1.06)	1.33 (1.28–1.38)	8.85 (8.27–9.47)	< 0.001
Married	11,255	0.78 (0.76–0.79)	Reference	Reference	
Formerly/ever married	2,684	1.53 (1.47–1.59)	1.97 (1.86–2.09)	1.37 (1.28–1.46)	< 0.001
**Region** ^†^					
North	1,988	0.87 (0.83–0.90)	1.08 (1.01–1.15)	1.38 (1.28–1.48)	< 0.001
Central	7,265	0.81 (0.79–0.83)	1.01 (0.95–1.07)	1.20 (1.12–1.28)	< 0.001
East	5,829	0.91 (0.88–0.93)	1.13 (1.06–1.21)	1.22 (1.13–1.31)	< 0.001
North-east	815	0.80 (0.74–0.85)	Reference	Reference	
West	4,407	1.07 (1.03–1.09)	1.33 (1.23–1.43)	1.67 (1.55–1.81)	< 0.001
South	6,405	1.13 (1.10–1.15)	1.41 (1.32–1.50)	1.66 (1.55–1.78)	< 0.001
**Religion** ^†^					
Hinduism	21,615	0.94 (.092–0.95)	0.92 (0.83–1.02)	0.93 (0.83–1.03)	0.175
Islam	3,462	0.89 (0.86–0.92)	0.87 (0.78–0.98)	0.84 (0.75–0.95)	0.006
Christianity	698	1.02 (0.94–1.09)	Reference	Reference	
Others	934	1.13 (1.06–1.20)	1.11 (0.98–1.27)	1.20 (1.32–1.51)	0.009
**Caste** ^†^					
Scheduled caste	6,164	0.99 (0.96–1.01)	1.13 (1.07–1.20)	1.27 (1.19–1.35)	< 0.001
Scheduled tribe	2,354	0.87 (0.83–0.90)	Reference	Reference	
Other backward class	11,361	0.95 (0.93–0.97)	1.08 (1.03–1.15)	1.35 (1.28–1.43)	< 0.001
Other	6,831	0.90 (0.88–0.92)	1.03 (0.97–1.10)	1.41 (1.32–1.19)	< 0.001
**Wealth index** ^†^					
Poorest	6,574	1.15 (1.12–1.18)	1.77 (1.67–1.88)	1.55 (1.43–1.68)	< 0.001
Poorer	6,044	1.06 (1.03–1.08)	1.62 (1.53–1.73)	1.38 (1.28–1.48)	< 0.001
Middle	5,595	0.98 (0.95–1.00)	1.51 (1.41–1.61)	1.24 (1.16–1.33)	< 0.001
Richer	4,789	0.84 (0.82–0.07)	1.29 (1.21–1.38)	1.11 (1.04–1.19)	0.002
Richest	3,707	0.65 (0.63–0.67)	Reference	Reference	
**Health insurance scheme (*****N*** = **2,829,625)**					
Absent	15,386	0.91 (0.89–0.93)	Reference	Reference	
Present	11,203	0.98 (0.95–1.00)	1.07 (1.03–1.10)	1.02 (0.98–1.06)	0.188
**BPL holder (*****N*** = **2,839,275)**^†^					
No	12,784	0.83 (0.81–0.84)	Reference	Reference	
Yes	13,877	1.07 (1.05–1.09)	1.30 (1.26–1.35)	1.08 (1.04–1.12)	< 0.001
**Treatment facility** ^†^					
Public facility	65,717	4.75 (4.71–4.78)	1.21 (1.05–1.40)	1.22 (1.05–1.42)	0.007
Private facility	60,767	4.30 (4.27–4.33)	1.00 (0.86–1.16)	1.08 (0.93–1.26)	0.281
NGO/Trust	662	4.98 (4.62–5.36)	1.30 (0.93–1.83)	1.34 (0.95–1.89)	0.089
Other	1,380	3.94 (3.74–4.15)	Reference	Reference	

PR, prevalence ratio; aPR, adjusted prevalence ratio; CI, confidence interval; BPL, below poverty level; NGO, non-government organization.

^*^Row percentage.

†p-value < 0.05.

In the published article, there was an error in Supplementary File 2. The prevalence of disability and distribution of different disability across sociodemographic was changed. The correct supplementary table and its caption appear below.

**Supplementary Table 1:**
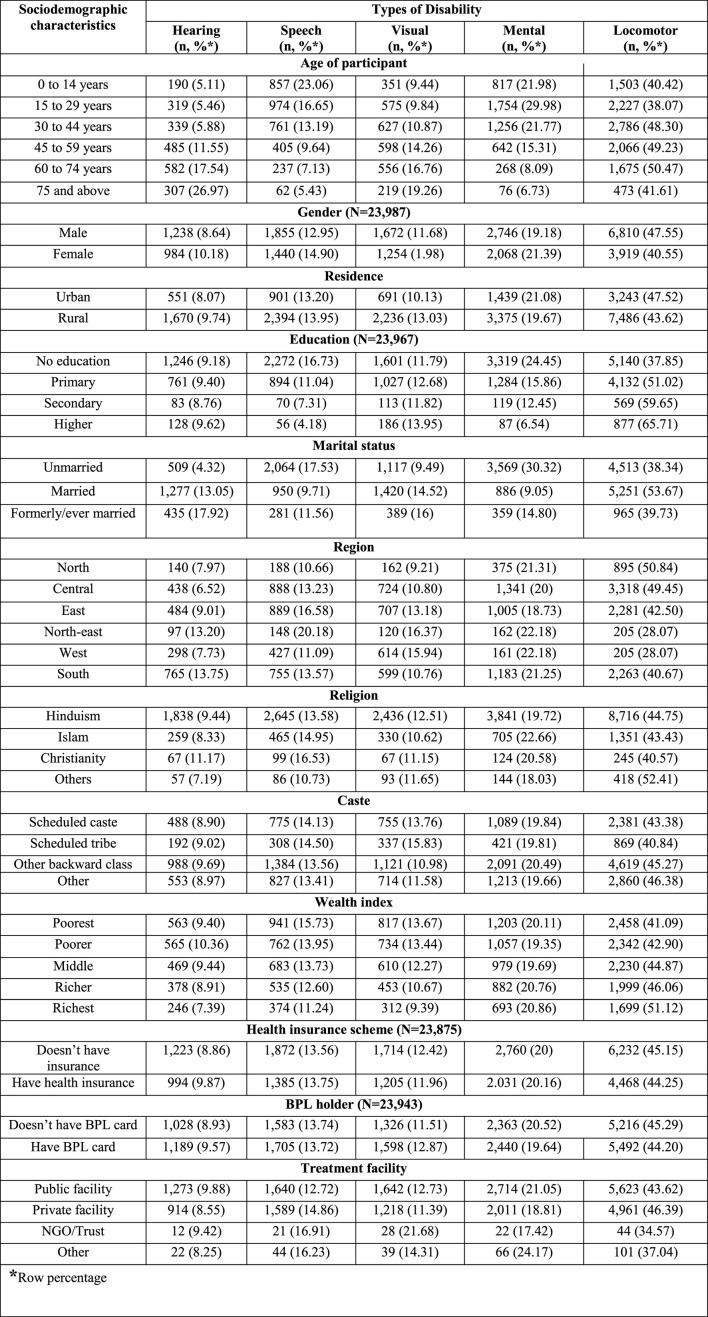
Pattern of disability across sociodemographic and health-seeking behavioral attributes in the study population covered in NFHS-5 (N = 23,988).

In the published article, errors were identified in the study findings presented in the **Abstract** section.

This sentence previously stated: “The overall prevalence of disability was 4.52% [(95% CI: 4.48–4.55), n = 1,28,528]. Locomotor disabilities accounted for 44.70% of all disabilities (n = 51,659), followed by mental disabilities (20.28%, n = 23,436). Age 75 years and above (vs. 0–14 years) [aPR: 2.65 (2.50–2.81)], male (vs. female) [aPR: 1.02 (1.0–1.04)], no education (vs. higher education) [aPR 1.62 (1.56–1.68)], unmarried (vs married) [aPR: 1.76 (1.70–1.82)], seeking the care of non-governmental organization (NGO) (vs. other) [aPR: 1.32 (1.13–1.55)] were significant independent determinants. The highest overall prevalence of locomotor was in Lakshadweep/UTs (8.88%) and Delhi (57.03%), respectively. Out of every hundred individuals in India, four have a disability.”

The corrected sentences appear below:

“The overall prevalence of disability was 0.93% [(95% CI: 0.92–0.95), *n* = 26,435] and 5.11% of households have one or more people with disability (PwD). Locomotor disabilities accounted for 44.73% of all disabilities (*n* = 10,730), followed by mental disabilities (20.07%, *n* = 4,814). Age 75 years and above (vs. 0–14 years) [aPR: 26.35 (23.63– 29.37)], male (vs. female) [aPR: 1.58 (1.52–1.64)], no education (vs. higher education) [aPR: 4.42 (4–4.87)], unmarried (vs. married) [aPR: 8.85 (8.27–9.47)], seeking care of non-governmental organization (NGO) (vs. other) [aPR: 1.34 (0.95–1.89)] were significant independent determinants. The highest overall prevalence of disability and locomotor was in Lakshadweep/UTs (1.68%) and Delhi (58.5%), respectively. Out of every hundred individuals in India, one has a disability, and five out of every hundred households have one or more people with a disability.”

In the published article, there was an error in the number of persons with disability. A correction has been made to **Methods**, *Statistical analysis*, paragraph one.

The sentence previously stated: “Therefore, they were excluded from the table of types of disabilities, giving a total number of persons with disability (*n* = 11,998).”

The corrected sentence appears below:

“Therefore, they were excluded from the table of types of disabilities, giving a total number of persons with disability (*n* = 2,447).”

In the published article, there were errors identified in the results and findings. A correction has been made to the entire **Results** section. The original reporting inaccurately reflected the overall prevalence of disability, prevalence across various age groups, comparisons between individuals aged 75 years and above and those aged 0–14 years, differences in prevalence between males and females, as well as variations based on education, marital status, region, caste, wealth quintile, and preferred health facility. Additionally, the distribution of disability percentages, the prevalence of locomotor disorders, and the descriptions of Figures 1A, 1B, 1C, and Figures 2A, 2B, 2C, and 2D were incorrectly presented.

The corrected **Results** appear below:

“The analysis includes a total of 2,843,917 respondents of all age groups. The respondents' mean (SD) age was 30.82 ± 20.62 years. Of the total, 26.92% were between the ages of 0 and 14 years (*n* = 765,602), 50.41% were females (*n* = 1,433,580), 75.83% belonged to rural residents (*n* = 2,156,633), and 49.99% were married (*n* = 1,421,809) (**Table 1**).

The overall prevalence of disability was 0.93% [(95% CI: 0.92–0.95), *n* = 26,435] and 5.11% of households have one or more people with disability (PwD) across all age groups in India. The prevalence was highest in the age group of 75 years and above at 1.96% (Table 2).

Respondents aged 75 years and above had twenty-six times [aPR: 26.35 (23.63–29.37)] the prevalence of disability compared with 0–14 years (Table 2). Disability was 58% more among males [aPR: 1.58 (1.52–1.64)] than females. Regarding education, disability was four times more common among those who didn't have any form of schooling [aPR: 4.42 (4–4.87)] in contrast to those who have completed higher education. Unmarried people had eight times more disability [aPR: 8.85 (8.27–9.47)] than married people. Respondents belonging to the west region [aPR: 1.67 (1.55–1.81)] have 67% more prevalence of disability compared with the north-east region. People from other backward castes had a 35% more burden of disability compared to people from scheduled tribe [aPR: 1.35 (1.28–1.43)]. Disability was 55% higher in the poorest wealth quintile [aPR: 1.55 (1.43–1.68)] than in most affluent. Individuals with disabilities favoured NGOs or Trust hospitals/clinics for medical care [aPR: 1.34 (0.95–1.89)] over visiting pharmacies or taking home treatment.

Of the total, locomotor disabilities accounted for 44.73% [(95% CI: 43.87–45.59), *n* = 10,730] followed by mental [20.07% (95% CI: 19.38–20.77), *n* = 4,814] and speech disabilities [13.74% (95% CI: 13.14–14.35, *n* = 3,295; Figure 1A]. The detailed prevalence of individual disabilities is given in Supplementary File 2. The ages of 60–74, 15–29, and 0–14 years had the highest burden of locomotor disability (50.47%), mental disability (29.98%), and speech disability (23.06%) respectively.

The preponderance of locomotor disability is highest among the 60–74 years age group. The prevalence pattern of various disabilities across the age groups is shown in Figure 1B.

The detailed prevalence pattern of various disabilities across educational status is shown in Figure 1C. Higher educational attainment is associated with a higher prevalence of locomotor and visual disabilities, as well as a lower prevalence of mental and speech disabilities.

Figures 2A–D shows the burden of disability and its pattern across the states and UTs of India. The overall disability distribution given in Figure 2A indicates that it is more prevalent in Lakshadweep, UT (1.68%), followed by Tamil Nadu (1.26%) and Karnataka (1.22%). In the present study, the regional disparities could be because of the fact that composition of the population and the individuals with disability varies in different states. So, the prevalence of disability varies in different states and found to be higher in Lakshadweep where the total population is less as compared with other states and UTs. For national representativeness, we have used the weighted values for data. Similarly, the prevalence of locomotor disability (Figure 2B) was highest in Delhi (58.5%), followed by Punjab (55.51%) and Madhya Pradesh (53.47%). Figure 2C shows the prevalence of mental disabilities, with the highest in Lakshadweep (41.24%), followed by Mizoram (38.12%) and Goa (37.1%). Figure 2D shows the highest prevalence of speech disability in Sikkim (37%), followed by Tripura (22.66%) and Jharkhand (22.12%).”

In the published article, errors were identified in the **Discussion** section. Corrections have been made to the following: The prevalence of disability concerning household prevalence in the first paragraph, the distribution of disability across states in India in the second paragraph, the comparison of disability distribution with other studies in the third paragraph, the discussion on marital status in the eighth paragraph, the topographical distribution of disability in the ninth paragraph, and the caste distribution in the tenth paragraph.

The original and corrected sentences appear below.

The first paragraph previously stated:

“The overall prevalence of disability in India based on secondary data analysis of the NFHS-5 survey (2019–21) was 4.52%. Locomotor disabilities accounted for 44.70% of all disabilities, followed by mental and speech disabilities. Age 75 years and above, male, no, unmarried, belonging to the west region, and non-governmental organization were significant independent determinants. The highest prevalence of locomotor, mental, and speech disability was in Delhi, Mizoram and Sikkim, respectively, whereas the overall prevalence was highest in Lakshadweep/UTs.”

The corrected paragraph appears below:

“The overall prevalence of disability in India based on secondary data analysis of the NFHS-5 survey (2019–2021) was 0.93% and 5.11% of households have one or more PwDs. Locomotor disabilities accounted for 44.73% of all disabilities, followed by mental and speech disabilities.” “The highest prevalence of locomotor, mental, and speech disability was in Delhi, Lakshadweep, and Sikkim, respectively, whereas the overall prevalence was highest in Lakshadweep/UTs.”

The first four sentences of the second paragraph previously stated:

“In the present study, the overall prevalence of disability was 4.52%. The result of this study is consistent with the findings of Myanmar (4.6%) and South Africa (4.9%) (28, 29). Our study reported a higher prevalence than in Zimbabwe (2.9%) and Cambodia (4%). Some countries reported a higher prevalence than the national average, including Jordan (13%) and Zimbabwe (7%) (30, 31).”

The corrected sentences appear below:

“In the present study, the prevalence of disability was found to be 0.93%, with 5.11% of households including one or more PwDs. While our study shows a notably lower overall prevalence of disability compared to countries like Myanmar (4.6%) and South Africa (4.9%), the household prevalence of PwDs is comparable or even higher. For instance, despite lower overall prevalence rates, the household prevalence in our study exceeds that reported in countries such as Zimbabwe (2.9%) and Cambodia (4%), and is similar to, or even higher than, the rates observed in countries like Jordan (13%) and Zimbabwe (7%) at the household level [30, 31]”

In the third paragraph the incorrect sentence previously stated:

“The present study highlights that locomotor disability was highest among those aged 0–14 years, which is in contrast to the study's findings, which suggest that it was higher among 40 years or older (34).”

The corrected sentence appear below:

“The present study highlights that locomotor disability was 286 highest among those aged 60–74 years [34].”

In the eighth paragraph the incorrect sentence previously stated:

“A study shows that unmarried people tend to suffer more from functional limitations, which is in line with our findings.”

The corrected sentence appears below:

“A study shows that formerly/ever-married and unmarried people tend to suffer more from functional limitations, which is in line with our findings.”

In the ninth paragraph the incorrect sentence previously stated:

“Topographically the western part was found to be a potential domain for disability in our study.”

The corrected sentence appears below:

“Topographically the southern part was found to be a potential domain for disability in our study.”

In the 10th paragraph, the incorrect sentence previously stated:

“The study conducted in Chennai among minorities suggested that rates of disability were higher among those belonging to Scheduled Tribes and Scheduled Castes (STs and SCs), which is in contrast with our study findings that is, disability was found to be more among those belonging to other backward class (OBCs) (56).”

The corrected sentence appears below:

“The study conducted in Chennai among minorities suggested that rates of disability were higher among those belonging to Scheduled Tribes and Scheduled Castes (STs and SCs). In contrast, our study found that disability was more prevalent among individuals belonging to Scheduled Castes (SCs) and Other Backward Classes (OBCs) [56].”

In the published article, errors were identified in the Conclusion section. Corrections have been made to the prevalence of disability.

The sentence previously stated: “The overall prevalence of disability in India is 4.5%.”

The corrected sentence appears below:

“The overall prevalence of disability in India is 0.93% and 5.11% of households have one or more people with disability (PwD).”

The authors apologize for these errors and state that this does not change the scientific conclusions of the article in any way. The original article has been updated.

