# Supplementary material for: Prevalence, pattern and determinants of disabilities in India: Insights from NFHS-5 (2019–21)
Source: Front Public Health. 2023 Feb 27;11:1036499. doi: 10.3389/fpubh.2023.1036499 (PMC10009251; doi:10.3389/fpubh.2023.1036499)
Supplement: Supplementary file 1 [file Table_1.DOCX]

**Operational definitions of various disabilities**

1. **Hearing**: For “Hearing”, considering functionality of both ears, individuals who could not hear at all and had hearing difficulty in daily communicative speech with or without a hearing aid were labelled with a hearing disability. Individuals with unilateral ear problems were not considered for inclusion in the hearing disability category.
2. **Speech:** Individuals were considered to have speech disabilities if they could not communicate properly due to a speech disability. Those who spoke only specific words, suffered articulation deformities, or stuttered were considered to have speech disability. People who stammered but spoke were not considered to have speech disability.
3. **Visual:** Individuals were considered to have "Visual" disabilities if they didn’t have light discernment or could not perceive light, and those who could not see correctly due to low or blurred vision. Despite using corrective remedies (such as spectacles or corrective lenses), people with low vision were classified as visually disabled. Persons who didn’t experience any trouble seeing after using corrective remedies and had normal vision in one eye (one-eyed people) were not presumed to be visually disabled.
4. **Mental:** Persons who lacked proper understanding for their age and had trouble carrying out regular activities such as communication (speech), self-care (brushing teeth, wearing clothes, taking a shower, taking food, personal hygiene, etc.), and those who had difficulty in speaking and understanding verbal and nonverbal messages were classified as “Mental” disability. Persons with intellectual disability were classified as mentally disabled. Due to age, people who exhibited mental exertion could not comprehend and relied on others for their day-to-day regime and were not deemed mentally disabled.
5. **Locomotor:** Persons with “Locomotor” disabilities were those who were deprived of the ability to use limb (didn’t have legs and hands, either unilateral or bilateral) or were paralysed and incapable of movement. Persons with loss or absence of part of a hand or leg, as well as those suffering from inactivity of a whole or part of the body due to amputation, paralysis, deformity (including hunch back, malformed spine, etc.), or dysfunction of limbs or joints, were considered to be disabled.
6. **Others:** Other form of disabilities was not specified under the heading "Others" in the categories of disabilities; therefore, they were excluded, keeping only the five main types.
