# Supplementary material for: Prevalence, pattern and determinants of disabilities in India: Insights from NFHS-5 (2019–21)
Source: Front Public Health. 2023 Feb 27;11:1036499. doi: 10.3389/fpubh.2023.1036499 (PMC10009251; doi:10.3389/fpubh.2023.1036499)
Supplement: Supplementary file 2 [file Table_2.docx]

**Supplementary table-1: Pattern of disability across sociodemographic and health-seeking behavioural attributes in the study population covered in NFHS-5 (N=23,988).**

| **Sociodemographic characteristics** | **Types of Disability** | | | | |
| --- | --- | --- | --- | --- | --- |
|  | **Hearing**  **(n, %*)** | **Speech**  **(n, %*)** | **Visual**  **(n, %*)** | **Mental**  **(n, %*)** | **Locomotor**  **(n, %*)** |
| **Age of participant** | | | | | |
| 0 to 14 years | 190 (5.11) | 857 (23.06) | 351 (9.44) | 817 (21.98) | 1,503 (40.42) |
| 15 to 29 years | 319 (5.46) | 974 (16.65) | 575 (9.84) | 1,754 (29.98) | 2,227 (38.07) |
| 30 to 44 years | 339 (5.88) | 761 (13.19) | 627 (10.87) | 1,256 (21.77) | 2,786 (48.30) |
| 45 to 59 years | 485 (11.55) | 405 (9.64) | 598 (14.26) | 642 (15.31) | 2,066 (49.23) |
| 60 to 74 years | 582 (17.54) | 237 (7.13) | 556 (16.76) | 268 (8.09) | 1,675 (50.47) |
| 75 and above | 307 (26.97) | 62 (5.43) | 219 (19.26) | 76 (6.73) | 473 (41.61) |
| **Gender (N=23,987)** | | | | | |
| Male | 1,238 (8.64) | 1,855 (12.95) | 1,672 (11.68) | 2,746 (19.18) | 6,810 (47.55) |
| Female | 984 (10.18) | 1,440 (14.90) | 1,254 (1.98) | 2,068 (21.39) | 3,919 (40.55) |
| **Residence** | | | | | |
| Urban | 551 (8.07) | 901 (13.20) | 691 (10.13) | 1,439 (21.08) | 3,243 (47.52) |
| Rural | 1,670 (9.74) | 2,394 (13.95) | 2,236 (13.03) | 3,375 (19.67) | 7,486 (43.62) |
| **Education (N=23,967)** | | | | | |
| No education | 1,246 (9.18) | 2,272 (16.73) | 1,601 (11.79) | 3,319 (24.45) | 5,140 (37.85) |
| Primary | 761 (9.40) | 894 (11.04) | 1,027 (12.68) | 1,284 (15.86) | 4,132 (51.02) |
| Secondary | 83 (8.76) | 70 (7.31) | 113 (11.82) | 119 (12.45) | 569 (59.65) |
| Higher | 128 (9.62) | 56 (4.18) | 186 (13.95) | 87 (6.54) | 877 (65.71) |
| **Marital status** | | | | | |
| Unmarried | 509 (4.32) | 2,064 (17.53) | 1,117 (9.49) | 3,569 (30.32) | 4,513 (38.34) |
| Married | 1,277 (13.05) | 950 (9.71) | 1,420 (14.52) | 886 (9.05) | 5,251 (53.67) |
| Formerly/ever married | 435 (17.92) | 281 (11.56) | 389 (16) | 359 (14.80) | 965 (39.73) |
| **Region** | | | | | |
| North | 140 (7.97) | 188 (10.66) | 162 (9.21) | 375 (21.31) | 895 (50.84) |
| Central | 438 (6.52) | 888 (13.23) | 724 (10.80) | 1,341 (20) | 3,318 (49.45) |
| East | 484 (9.01) | 889 (16.58) | 707 (13.18) | 1,005 (18.73) | 2,281 (42.50) |
| North-east | 97 (13.20) | 148 (20.18) | 120 (16.37) | 162 (22.18) | 205 (28.07) |
| West | 298 (7.73) | 427 (11.09) | 614 (15.94) | 161 (22.18) | 205 (28.07) |
| South | 765 (13.75) | 755 (13.57) | 599 (10.76) | 1,183 (21.25) | 2,263 (40.67) |
| **Religion** | | | | | |
| Hinduism | 1,838 (9.44) | 2,645 (13.58) | 2,436 (12.51) | 3,841 (19.72) | 8,716 (44.75) |
| Islam | 259 (8.33) | 465 (14.95) | 330 (10.62) | 705 (22.66) | 1,351 (43.43) |
| Christianity | 67 (11.17) | 99 (16.53) | 67 (11.15) | 124 (20.58) | 245 (40.57) |
| Others | 57 (7.19) | 86 (10.73) | 93 (11.65) | 144 (18.03) | 418 (52.41) |
| **Caste** | | | | | |
| Scheduled caste | 488 (8.90) | 775 (14.13) | 755 (13.76) | 1,089 (19.84) | 2,381 (43.38) |
| Scheduled tribe | 192 (9.02) | 308 (14.50) | 337 (15.83) | 421 (19.81) | 869 (40.84) |
| Other backward class | 988 (9.69) | 1,384 (13.56) | 1,121 (10.98) | 2,091 (20.49) | 4,619 (45.27) |
| Other | 553 (8.97) | 827 (13.41) | 714 (11.58) | 1,213 (19.66) | 2,860 (46.38) |
| **Wealth index** | | | | | |
| Poorest | 563 (9.40) | 941 (15.73) | 817 (13.67) | 1,203 (20.11) | 2,458 (41.09) |
| Poorer | 565 (10.36) | 762 (13.95) | 734 (13.44) | 1,057 (19.35) | 2,342 (42.90) |
| Middle | 469 (9.44) | 683 (13.73) | 610 (12.27) | 979 (19.69) | 2,230 (44.87) |
| Richer | 378 (8.91) | 535 (12.60) | 453 (10.67) | 882 (20.76) | 1,999 (46.06) |
| Richest | 246 (7.39) | 374 (11.24) | 312 (9.39) | 693 (20.86) | 1,699 (51.12) |
| **Health insurance scheme (N=23,875)** | | | | | |
| Doesn’t have insurance | 1,223 (8.86) | 1,872 (13.56) | 1,714 (12.42) | 2,760 (20) | 6,232 (45.15) |
| Have health insurance | 994 (9.87) | 1,385 (13.75) | 1,205 (11.96) | 2.031 (20.16) | 4,468 (44.25) |
| **BPL holder (N=23,943)** | | | | | |
| Doesn’t have BPL card | 1,028 (8.93) | 1,583 (13.74) | 1,326 (11.51) | 2,363 (20.52) | 5,216 (45.29) |
| Have BPL card | 1,189 (9.57) | 1,705 (13.72) | 1,598 (12.87) | 2,440 (19.64) | 5,492 (44.20) |
| **Treatment facility** | | | | | |
| Public facility | 1,273 (9.88) | 1,640 (12.72) | 1,642 (12.73) | 2,714 (21.05) | 5,623 (43.62) |
| Private facility | 914 (8.55) | 1,589 (14.86) | 1,218 (11.39) | 2,011 (18.81) | 4,961 (46.39) |
| NGO/Trust | 12 (9.42) | 21 (16.91) | 28 (21.68) | 22 (17.42) | 44 (34.57) |
| Other | 22 (8.25) | 44 (16.23) | 39 (14.31) | 66 (24.17) | 101 (37.04) |
| *****Row percentage | | | | | |
